# A Natural Bio-Stimulant Consisting of a Mixture of Fish Protein Hydrolysates and Kelp Extract Enhances the Physiological, Biochemical and Growth Responses of Spinach under Different Water Levels

**DOI:** 10.3390/plants11233374

**Published:** 2022-12-05

**Authors:** Pule Clement Liatile, Gerhard Potgieter, Makoena Joyce Moloi

**Affiliations:** Department of Plant Sciences-Botany Division, University of the Free State, 205 Nelson Mandela Drive, Park West, Bloemfontein 9301, South Africa

**Keywords:** bio-stimulants, chlorophyll-*a* fluorescence, drought stress, normalised difference vegetation index, osmoprotectants, photosynthesis, *Spinacia oleracea*, stomatal conductance, vegetative growth

## Abstract

Spinach (*Spinacia oleracea* L.) is a highly nutritious, desirable green leafy vegetable, which is less tolerant to drought. This study was conducted to establish the impact of a natural bio-stimulant consisting of a mixture of fish protein hydrolysates and kelp extract (trade name, *Xcell Boost*) on the physiological and biochemical responses as well as vegetative growth of spinach (*Spinacia oleracea* L.) under different water levels (100% (full irrigation), 50% (mild drought stress) and 30% (severe drought stress) water holding capacity). Bio-stimulant application at any strength (single, BX1 or double, BX2) had no effect on the photochemical reactions. The application of bio-stimulant at double strength concentration (BX2) increased the chlorophyll and carotenoid contents, as well as the activities of antioxidative enzymes, ascorbate peroxidase (APX) and guaiacol peroxidase (GPX), under drought stress. Application at single strength (BX1) increased the normalised difference vegetation index (NDVI), stomatal conductance, accumulation of osmoprotectants (proline and total soluble sugars) and reduced electrolyte leakage under drought stress. Furthermore, bio-stimulant applications at either concentration induced remarkable increases in plant height, leaf area, stem dry weight, root length and root moisture. Under BX2, APX and stomatal conductance positively correlated with stem dry weight, while root length positively correlated with total chlorophyll content. These results show that *Xcell Boost* is a highly advantageous bio-stimulant for increasing the tolerance of spinach to drought stress, which can most likely benefit other crops grown in semi-arid and arid areas.

## 1. Introduction

Spinach (*Spinacia oleracea* L.), belonging to the Amaranthaceae family, is a highly nutritious and desirable green leafy vegetable for consumers in different parts of the globe [1,2,3], including South Africa. Drought is a major and widespread stress factor for plants in most parts of the world [4], particularly in arid and semi-arid regions, such as South Africa [5]. Most of the leafy vegetables, including spinach, are less tolerant to drought [6,7].

At the physiological level, drought stress may damage the photosynthesis apparatus and cell membranes, which interfere with many metabolic processes [8], and ultimately affect morphological responses [9]. For instance, drought stress can damage the reaction centres of photosystem ꓲꓲ and inhibit photochemical activity, which decrease chlorophyll-*a* fluorescence [10]. Chlorophyll-*a* fluorescence parameters, such as maximum quantum efficiency of photosystem II reaction centres (*Fv*/*Fm*), performance index absorbance (PI_ABS_) and total performance index (PI_Total_), can be used to explore the photosynthetic apparatus under different environmental conditions [11,12,13]. Shin et al. [14] found that chlorophyll-a fluorescence parameters were significantly reduced under severe drought stress in lettuce (*Lactuca sativa* L.). In contrast, Xu and Leskovar [15] showed that *Fv/Fm* was not affected by water stress in spinach (*Spinacia oleracea* L.). This shows that the effect of drought stress on vegetable crops depends on the plant species or vegetable type [16]. Drought stress may also reduce the accumulation of chlorophyll pigments, decrease the leaf area and consequently inhibit photosynthesis per unit area [17]. Ekinci et al. [18] confirmed that chlorophyll content in spinach leaves decreased under different irrigation levels. However, Reyes et al. [19] stated that if the plants are not subjected to intense drought stress, their chlorophyll content will not be affected, showing the significance of drought intensity on plants, especially spinach.

One of the earliest plant responses to limited water availability includes stomatal closure, which subsequently leads to a decrease in photosynthesis capacity [20] while increasing the water use efficiency [21]. Stomatal conductance can be used to estimate the plant yield of certain C_3_ crops under different water levels, suggesting that higher stomatal conductance can be correlated with high yield [22]. Stomatal closure due to drought stress causes excitation or partial reduction in molecular oxygen (O_2_), which may lead to overproduction of reactive oxygen species (ROS), a condition called oxidative burst [23,24]. Oxidative burst impairs metabolic functions and causes irreversible damage to the plant [25]. It inactivates enzymes and damages plant cell membranes, resulting in increased solutes and ion (electrolyte) leakage [26,27].

To avoid drought-stress-induced oxidative burst, plants may produce antioxidative systems, including enzymatic antioxidants, such as ascorbate peroxidase (APX), superoxide dismutase (SOD), guaiacol peroxidase (GPX) and glutathione reductase (GR), or non-enzymatic antioxidants, such as ascorbic acid (AA), glutathione (GSH) and tocopherol (TOC) [25,28]. Several studies have reported that an increase in osmolytes, total soluble sugars, and enzymatic and non-enzymatic antioxidants in different vegetable crops under abiotic stress is also associated with drought tolerance [29,30]. In addition, Moloi and van der Merwe [31] reported an increase in free proline content, total soluble sugars and enzymatic antioxidants in vegetable-type soybean cultivars under water stress. However, the accumulation of osmolytes, soluble sugars and antioxidants is not only observed in water-stress-tolerant plants but also in water-stress-sensitive vegetable crops, such as spinach [32].

Other alternatives to alleviate water stress in plants involve the application of bio-stimulants [33]. Bio-stimulants are available in liquid extracts, soluble powder and granular forms and can be applied as both foliar or soil applications [34]. In horticulture, bio-stimulants are categorised into four major groups: (1) humic substances (HS), (2) protein hydrolysate and amino acid formulations (AA), (3) seaweed extract (SWE) and (4) plant-growth-promoting micro-organisms. These categories are based on the origin or their source and the effect of each bio-stimulant on root growth and nutrient uptake, not on their chemical composition [35]. Seaweed extract, *Ascophyllum nodosum*, was used as a bio-stimulant to improve the vegetative parameters [18] and physiological parameters [15] of spinach under drought stress. A few studies showed that seaweed extracts have a positive effect on the antioxidant activity in plants under stress [36,37]. Furthermore, foliar application of plant-derived protein hydrolysates improved spinach antioxidant activity and nutrient content under nutrient deficiency [38]. Lastly, animal-derived protein hydrolysates from chicken feathers [39] and fish by-products [40,41] were reported to enhance the enzyme activities and content of free proline under optimal conditions. In addition to increasing crop yields, natural bio-stimulants can also influence the nutritional quality of crops. In spinach, the application of seaweed extract increased the primary and secondary metabolism, which resulted in increased nutrient use efficiency, thereby increasing nutritional quality [42].

There are no studies on the effects of combined protein hydrolysate and seaweed extracts on the physiological and biochemical responses of spinach under drought stress. The application of such a natural bio-stimulant could enhance drought stress tolerance in spinach, which could be a possible eco-friendly solution under the current climatic changes. Therefore, this study aimed at investigating the effects of a new bio-stimulant, *Xcell Boost* (containing hydrolysed fish protein (HFP) and Kelp extract), on the photosynthetic efficiency, biochemical and yield responses of spinach growing under different water levels. The relationships between the growth/yield and photosynthesis/biochemical parameters of bio-stimulant-treated spinach will be established under drought. This will determine whether an improvement of the physiology/biochemistry of spinach after bio-stimulant treatment translates into better yield, thereby combating the negative effects of drought stress on spinach.

## 2. Results

### 2.1. Photosynthesis Parameters

The *Fv/Fm*, PI_ABS_ and PI_Total_ of spinach grown under different water levels and *Xcell Boost* concentrations are given in Table 1. The *Fv/Fm* for 50% and 30% WHC remained unchanged (>0.80) compared to 100% WHC, with no substantial effect after *Xcell Boost* applications (means followed by the same letters, *p* > 0.05). Compared to the 100% WHC treatment, the 50% and 30% WHC increased PI_ABS_ (23% and 14% increase, respectively). Application of *Xcell Boost* had insignificant effect on the PI_ABS_ under well-watered and water-limited conditions (*p* > 0.05). Similarly, PI_Total_ increased under stress. Application of different concentrations of the bio-stimulant led to significant decreases in the PI_Total_ under 50% WHC.

**Figure 1:**
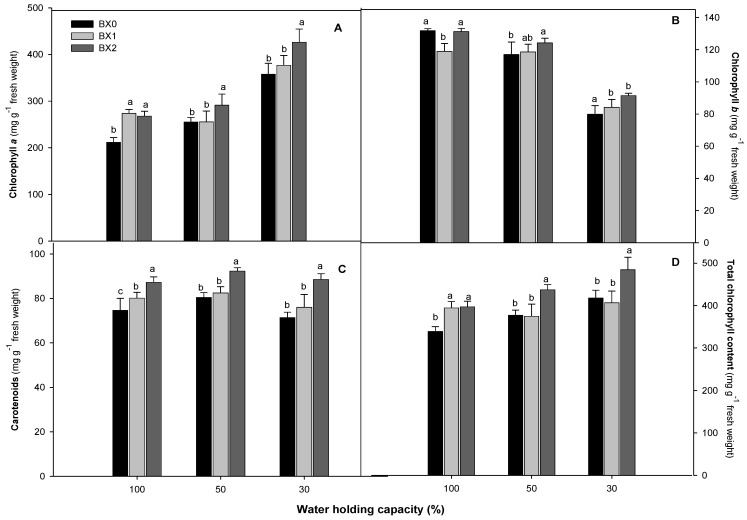
Chlorophyll a (**A**), chlorophyll b (**B**), carotenoid (**C**) and total chlorophyll content (**D**) of spinach grown under different water and *Xcell Boost* levels. The values are means of four replicates ± standard error. Letters on top of bars indicate significant differences within each water treatment at *p* ≤ 0.05. BX0 represents no bio-stimulant, while BX1 represents single concentration and BX2 double concentration of the bio-stimulant.

Chlorophyll *a* content increased with drought stress application. The treatment of plants with BX2 significantly (*p* ≤ 0.05) increased chlorophyll *a* under 50% (from 255 to 291) and 30% (from 358 to 426) WHC (Figure 1A). Chlorophyll *b* content declined with water deficiency treatments. Although the BX1 treatment reduced the chlorophyll *b* content under 100% WHC, it led to significant increases under 30% WHC (356 to 377). The BX2 treatment induced the highest chlorophyll *b* content under both water deficiency treatments (Figure 1B). The carotenoid content was slightly reduced under 30% WHC. The BX2 led to a significant (*p* ≤ 0.05) increase in carotenoid content at 50% and 30% WHC (13% and 19%) (Figure 1C). The total chlorophyll content increased with the increase in water deficiency. The BX2 treatment induced significant increases (*p* ≤ 0.05) in the total chlorophyll content for the 50% WHC (14% increase) and 30% WHC (14% increase) treatments (Figure 1D).

**Figure 2:**
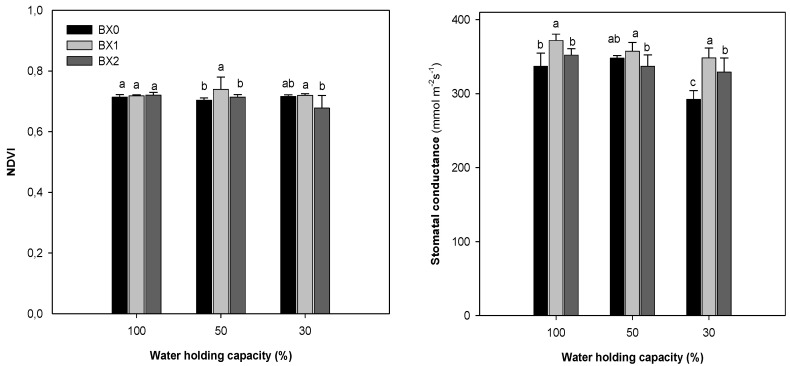
Normalised difference vegetation index (NDVI) and stomatal conductance of spinach grown under different water and *Xcell Boost* levels. The values are means of four replicates ± standard error. Letters on top of bars indicate significant differences within each water treatment at *p* ≤ 0.05. BX0 represents no bio-stimulant, while BX1 represents single concentration and BX2 double concentration of the bio-stimulant.

The NDVI did not change with changing soil water content, as it was above 0.6 for all treatments. Under optimal watering, *Xcell Boost* did not have any effect on the NDVI values. Application of BX1 on the plant under 50% WHC treatment led to significantly increased NDVI. A reduction in stomatal conductance was evident under the 30% WHC treatment (13% reduction). Application of BX1 showed the highest significant (*p* ≤ 0.05) increase in stomatal conductance under 30% WHC (292 to 348). The BX2 application also induced a significant (*p* ≤ 0.05) increase in this parameter (292 to 329), but the increase was lower than that of the BX1 treatment (Figure 2).

### 2.2. Osmolytes and Electrolyte Leakage

**Figure 3:**
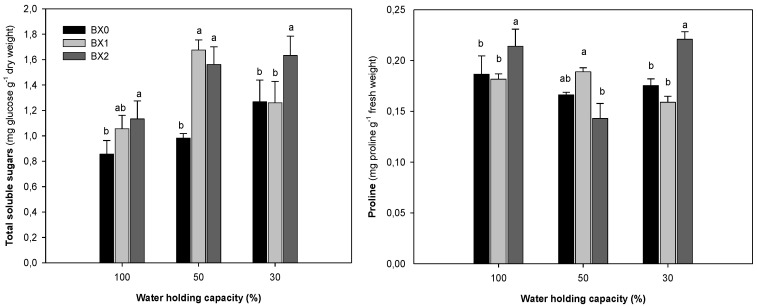
Total soluble sugar content and proline content of spinach grown under different water and *Xcell Boost* levels. The values are means of four replicates ± standard error. Letters on top of bars indicate significant differences within each water treatment at *p* ≤ 0.05. BX0 represents no bio-stimulant, while BX1 represents single concentration and BX2 double concentration of the bio-stimulant.

The total soluble sugars (TSS) increased with the severity of drought. BX1 induced significant increase in the TSS content under 50% WHC only. The BX2 treatment induced substantial (*p* ≤ 0.05) increases in the TSS accumulation under all water levels, with 50 and 30% WHC showing the highest increases (37% and 22%). Water deficit reduced the proline content slightly. The BX2 treatment induced significantly high levels of proline (0.175 to 0.221) at 30% WHC (Figure 3).

**Figure 4:**
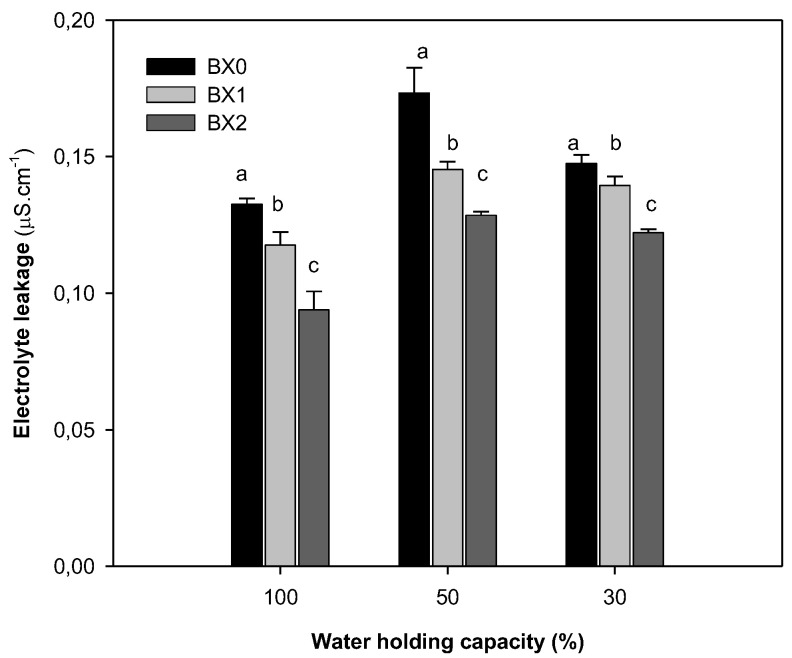
Electrolyte leakage content of spinach grown under different water and *Xcell Boost* levels. The values are means of four replicates ± standard error. Letters on top of bars indicate significant differences within each water treatment at *p* ≤ 0.05. BX0 represents no bio-stimulant, while BX1 represents single concentration and BX2 double concentration of the bio-stimulant.

Water deficiency stress increased the electrolyte leakage. The application of *Xcell Boost* significantly prevented the electrolyte leakage levels across all treatments, with the BX2 treatment showing the lowest electrolyte leakage (Figure 4).

### 2.3. Antioxidant Enzyme Activities

**Figure 5:**
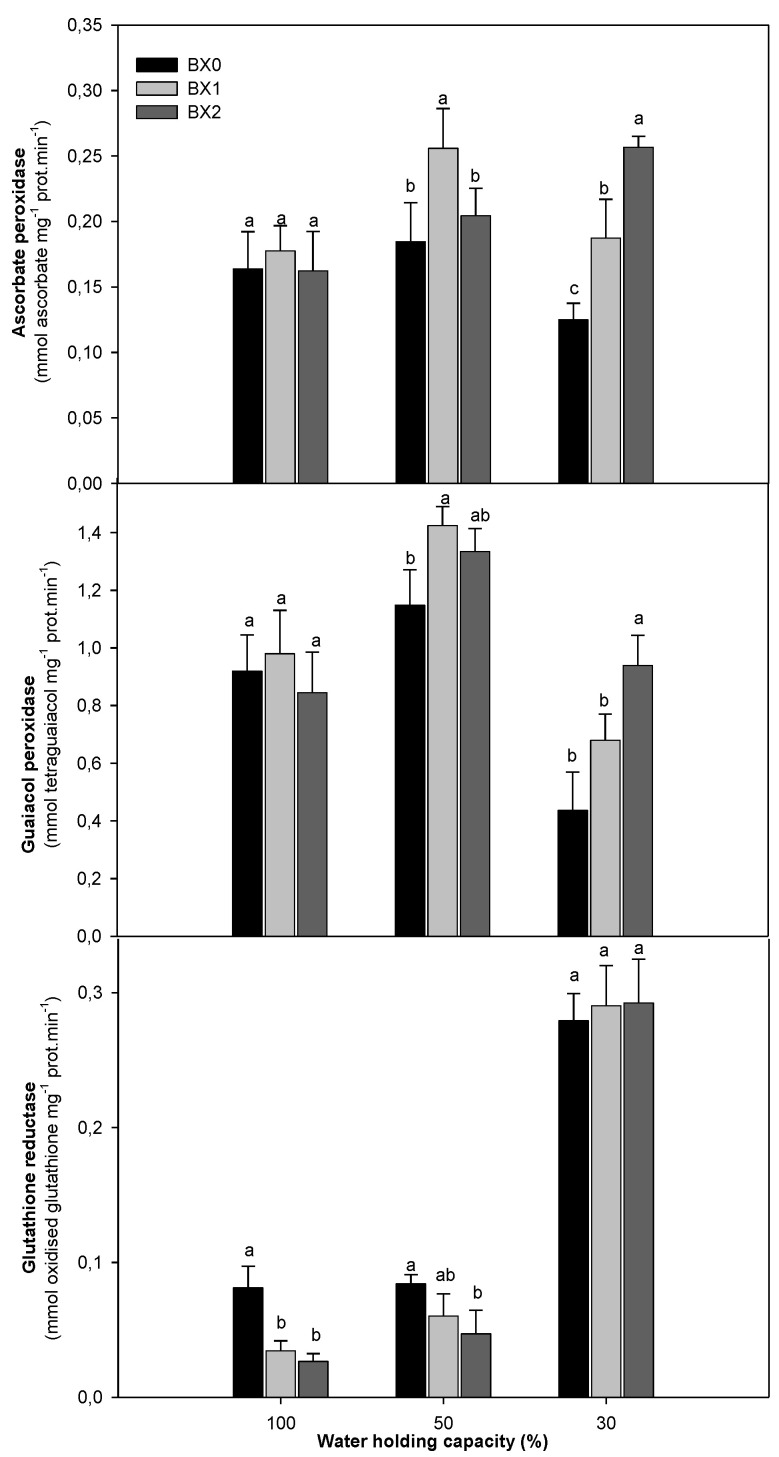
Ascorbate peroxidase activity, guaiacol peroxidase activity and glutathione reductase activity of spinach grown under different water and *Xcell Boost* levels. The values are means of four replicates ± standard error. Letters on top of bars indicate significant differences within each water treatment at *p* ≤ 0.05. BX0 represents no bio-stimulant, while BX1 represents single concentration and BX2 double concentration of the bio-stimulant.

Severe water deficiency (30% WHC) significantly reduced the APX activity. The BX1 application increased APX activity under both water deficiency levels (0.185 to 0.256 and 0.125 to 0.187 for 50% WHC and 30% WHC, respectively). The BX2 treatment induced a significant (*p* ≤ 0.05) increase (21%) under 30% WHC. The GPX activity was substantially reduced under 30% WHC (53%). The two *Xcell Boost* concentrations had no significant effect on the activity of this enzyme under optimal water treatment but induced it under 50% and 30% WHC. The BX2 treatment induced the highest significant (*p* ≤ 0.05) increase at 30% WHC (53.5%). The activity of GR increased with an increase in water deficiency. Application of *Xcell Boost* significantly decreased the GR activity at 100% and 50% WHC but was insignificant under 30% WHC (Figure 5).

### 2.4. Vegetative Growth Responses

Severe drought stress (30% WHC) reduced the plant height (22%). The application of BX1 significantly increased plant height under 30% WHC (18.4 to 21.6). The root length was reduced under the 30% WHC treatment. The application of *Xcell Boost* significantly increased the root length under 30% WHC, with the BX2 showing the highest significant increase (25%) in root length (Table 2). The leaf surface area was substantially reduced under the 50% and 30% WHC treatments. The BX1 and BX2 treatments stimulated significant increases in the leaf surface area under 50% WHC. Under 30% WHC, only the BX2 treatment showed a significant (*p* ≤ 0.05) increase in the leaf surface area (723 to 843). There were insignificant differences in the number of leaves for *Xcell Boost* concentrations under all water treatments. The 30% WHC treatment reduced the leaf dry weight substantially (24%). The addition of the *Xcell Boost* at both concentrations induced the leaf dry weights, but not significantly, for both 50% and 30% WHC. The stem dry weight was reduced under both water deficiencies. *Xcell Boost* had no effect on this parameter, except for BX2, which significantly increased the stem dry weight (3.837 to 5.239) under 50% WHC. The application of *Xcell Boost* had an insignificant effect on root dry weight under all water treatments. There was a decrease in the relative water content (RWC) under water-deficient conditions. The BX1 treatment induced RWC under drought stress conditions. The BX2 increased RWC significantly (72.73 to 78.93 and 68.35 to 76.49 under 50% and 30% WHC, respectively) to the levels close to 100% WHC. Stem moisture content remained unchanged under water-deficiency stress compared to the optimal water treatment. All treatments had a moisture content above 85%. The application of *Xcell Boost* had insignificant effects on the stem moisture content across all the treatments. Root moisture content decreased with an increase in the severity of water stress. The application of BX1 concentration slightly increased root moisture under 100% WHC but significantly reduced the root moisture content under 50% WHC (7% decrease) and 30% WHC (19% decrease). The concentration of BX2 reduced root moisture content under 100% WHC but increased root moisture content under 30% WHC (56.22 to 61.82).

### 2.5. The Correlations between the Photosynthetic, Vegetative and Biochemical Responses of Xcell Boost Treated Spinach under Severe Water Deficiency

The application of BX1 under severe water deficiency indicated significantly negative correlations between leaf dry weight (LDW) and GPX, stem dry weight (SDW) and leaf moisture content (MC leaf), PI_Total_ with carotenes and chlorophyll *a* (Chl-*a*). Significantly positive correlations for this treatment included LDW and leaf number, stomatal conductance (SC) with APX and GR, PI_ABS_ and proline, MC root and PI_Total_. In contrast, the application of BX2 under severe water deficiency indicated a high number of significantly positive correlations and a few negative correlations (*p* < 0.05). The RWC negatively correlated with SC and SDW, while GPX negatively correlated with plant height. PI_ABS_ negatively correlated with chlorophyll *b* (Chl-*b*). Significantly positive correlations (*p* < 0.05) between the root length (RL) and MC leaf, total chlorophyll (chl-total) and LDW were observed. Other significantly positive correlations under BX2 were between stomatal conductance (SC) and SDW, as well as APX, GR and NDVI (Table 3).

## 3. Discussion

Drought is a significant abiotic stress factor, which affects plant productivity and yield because it interferes with numerous metabolic systems and reduces photosynthesis [43]. When plants are subjected to abiotic conditions, such as drought, the *Fv/Fm* ratio, which represents the quantum efficiency of PSII, is used to assess plant health and detect changes in the photosynthetic system [44,45]. In this study, however, water deficiency and *Xcell Boost* application had no effect on PSII quantum efficiency, indicating that it is not a sensitive parameter under water deficiency in spinach. Although other chlorophyll *a* fluorescence parameters, such as PI_ABS_ and PI_Total_, increased with drought stress (50% and 30% WHC), the application of *Xcell Boost* did not further improve their functioning. The parameters of chlorophyll *a* fluorescence can be combined with other measurements to produce a comprehensive understanding of the photosynthetic system [44,46]. Total chlorophyll and chlorophyll *a* content increased with the severity of water deficiency, and *Xcell Boost* treatment (BX2, double strength) was able to induce the highest increase under drought stress conditions. This implies that *Xcell Boost* application could increase the amount of light energy captured by the plants under drought stress, thereby increasing the photosynthetic capacity of spinach [47]. On the contrary, drought stress decreased the chlorophyll content in maize, apples and grapes [17,48,49], respectively. Although the amount of chlorophyll *b* decreased with the severity of drought stress in spinach, the *Xcell Boost* treatment improved the chlorophyll *b* content, with BX2 showing more efficiency. The protein hydrolysates contained in *Xcell Boost* could increase the content of chlorophyll *b,* an accessory pigment that transports light energy to chlorophyll *a* [50,51], thereby maximising the photosynthesis capacity of spinach. The NDVI is a measure of vegetation “greenness” and can be associated with potentially high chlorophyll content. Plants with NDVI values between 0.6 and 0.9 are considered healthy [52,53]. Crusiol et al. [54] found that the NDVI values of two soybean cultivars (drought-sensitive cultivar and less drought-sensitive cultivar) with differing responses to drought were comparable with only minor changes seen under water deficiency during the vegetative growing period. However, it was discovered that the NDVI values of the cultivars that were less sensitive to drought during the reproductive stage were greater than those of the cultivars that were more sensitive to drought. In this investigation, the application of *Xcell Boost* treatment (BX1, single strength) considerably raised the NDVI values under water deficiency stress. When screening for drought-tolerant sorghum cultivars, Devnarain et al. [55] reported that the carotenoid content was not significantly reduced under water deficiency stress. Similarly, the findings of the current study indicated that the carotenoid content was slightly lower under severe water deficiency stress compared to the optimal water treatment. However, when treated with *Xcell Boost* (BX2), the carotenoid increased substantially to the same level as non-stressed control plants, further demonstrating the significance of *Xcell Boost* in drought tolerance of spinach.

One of the earliest and most typical responses of plants to water stress is a decrease in stomatal conductance [56,57]. According to Xu and Leskovar [15], stomatal conductance decreased in cabbage during mild water stress. In agreement, the stomatal conductance was significantly reduced (13%) under severe drought stress in this study. *Xcell Boost* significantly enhanced the stomatal conductance of spinach under severe drought stress. *Xcell Boost* clearly showed bio-stimulatory properties by reducing the negative impact of water stress deficiency, enabling plants to enhance their stomatal conductance (increased CO_2_ uptake), which potentially resulted in higher photosynthetic capacity. Similarly, Ekinci et al. [18] reported that application of a liquid organic amendment containing amino acids and organic matter induced the stomatal conductance in spinach under different irrigation levels.

To increase their osmotic potential and improve water retention, plants respond to water stress by increasing the synthesis of soluble sugars and other osmolytes [58,59]. Our findings are partially in accordance with previous results that show that drought stress increases soluble sugars and free proline content in plants [60,61,62]. Accumulation of the total soluble sugars (TSS) significantly increased with the severity of water deficiency, while proline content decreased. Proline plays a key role in plant defence as an osmoprotectant and can function as an antioxidant [29,63]. Proline is also essential for plant metabolism [60]; thus, its low accumulation might be due to proline catabolism during drought stress in the proline metabolism. Application of *Xcell Boost* significantly increased TSS and proline content under water deficiency treatments, showing the importance of this bio-stimulant in the drought tolerance responses of spinach.

Drought stress results in excessive production of ROS, causing oxidative stress. Plasma membrane damage from oxidative stress leads to increased solute and electrolyte (ion) leakage [26]. Ekinci et al. [18] reported that EL significantly increased with a decrease in irrigation amounts. The level of EL significantly increased with an increase in water deficiency stress, suggesting more membrane damage. However, the application of *Xcell Boost* (Bx2) significantly reduced EL across all treatments, indicating its bio-stimulatory properties by making spinach more tolerant under drought stress. In agreement, Patel et al. [64] reported that a seaweed (*Kappaphycus alvarezii*) extract application reduced EL and lipid peroxidation (malondialdehyde) under saline and drought stress in wheat. To reduce the EL and prevent oxidative stress, plants need to possess active ROS scavenging systems. This, however, depends on the plant species and even the cultivars [31]. Sahin et al. [65] found that the antioxidative enzyme activities of cabbage decreased with an increase in drought. In this study, the activities of APX and GPX were substantially inhibited under severe water deficiency stress. Although the application of *Xcell Boost* had insignificant effect on these enzymes under optimal water treatment, it was highly effective under drought stress, with BX2 showing the most significant increase. These findings agree with Trivedi et al. [66], who discovered that the *Kappaphycus alvarezii* seaweed extract induces the antioxidative responses of maize under drought stress. This suggests that *Xcell Boost* may enhance spinach’s antioxidant defences under water deficiency stress. The strong, positive correlation between the APX and stomatal conductance further indicates that *Xcell Boost* plays an important role in the improvement of plant performance under drought stress. Contrarily, under drought stress, the glutathione reductase (GR) activity increased (3-fold), suggesting the increased ROS scavenging ability of spinach. However, the treatment with *Xcell Boost* (regardless of concentration) had no effect on GR activity (the activity remained the same as that of the control), indicating that this enzyme was not responsible for the observed decrease in EL under treatment with this bio-stimulant.

The leaf RWC and plant moisture content are useful drought stress indicators, which are closely associated with the leaf water status and cell turgor, which is essential in understanding plant growth (productivity) and development [67]. Xu and Leskovar [15] reported that RWC was significantly reduced under mild water stress in spinach, and the application of an *Ascophyllum nodosum* seaweed extract improved RWC. Similarly, in this study, RWC decreased under water-deficient conditions. The application of *Xcell Boost* significantly increased RWC under drought stress conditions, especially at high concentrations (BX2). This increase could possibly be a result of increased TSS and proline under BX2, which are involved in osmoregulation [63], thereby improving the water content of the leaves. Stem moisture and root moisture content decreased with the severity of water stress. The application of *Xcell* Boost (BX2) improved the root moisture content under water deficiency stress. Plant biomass (plant height, root length, leaf and stem dry weights) was negatively affected by water-deficient conditions, except for root dry weight and a number of leaves that were not significantly affected. Luoh et al. [68] and Maseko et al. [6] found comparable outcomes with leafy vegetables under water deficit conditions. Furthermore, the leaf surface area was reduced under water-deficient stress. Previous research on spinach found that abiotic stressors, including salinity, water, and nutrient deficiency [7,32,69], limit leaf growth. Under optimal irrigation, BX2 significantly increased leaf dry weight, stem dry weight under mild conditions and induced root dry weight, but not significantly. Application of different concentrations of *Xcell Boost* under severe drought stress significantly increased plant height (BX1), root length and leaf surface area (BX2). Xu and Leskovar [15] reported that the application of an *Ascophyllum nodosum* seaweed extract improved spinach leaf growth under drought stress.

## 4. Materials and Methods

### 4.1. Plant Material and Experimental Setup

Spinach seeds (*Spinacia oleracea* L.) cv., Fordhook Giant, were germinated in seedling trays filled with a seedling mix, Hygromix (Hygrotech (Pty) Ltd., Pretoria, South Africa), and watered daily. Fourteen days after germination, one seedling was transplanted into a pot (7 L capacity, 25 cm diameter and 20 cm height) containing a mixture of coarse gravel (590 g placed on nylon mesh at the base) and loamy-sandy red soil (7000 g at the top). The study was conducted at the greenhouse facility of the University of the Free State, Bloemfontein (29°6′31.94″ S, 26°11′18.95″ E), at 25 °C (day) and 18 °C (night) temperatures, under natural light. The experimental layout was a split-plot design with complete randomisation and four replications. The main plot was water treatment, and the subplot was *Xcell Boost* treatment. There were three water treatment (30% (severe drought), 50% (mild drought) and 100% (control) water levels and three treatments of *Xcell Boost* (the control (no bio-stimulant, BX0), single (BX1) and double (BX2) concentration of bio-stimulant).

Water deficiency (drought) stress was initiated four weeks after transplantation. Pots were irrigated to field capacity/100% water holding capacity (WHC); then, irrigation was withdrawn to the appropriate water level (50% and 30% WHC). The moisture in the pot plants was maintained with daily hand irrigation using tap water by weighing each pot before irrigation.

*Xcell Boost* (a mixture of 100% Hydrolysed Fish Protein (HFP) and 100% Kelp (*Ecklonia maxima*)) prepared by Introlab Pty., was applied every third week (i.e., 21 days) after irrigation according to the manufacturer’s guidelines [70]. Two different dosages (single dosage; BX1) as well as a double dosage (BX2) were prepared by adding 2 mL HFP and 0.5 mL Kelp stock solutions to 247.5 mL distilled water to prepare a 250 mL solution and 4 mL HFP and 1 mL Kelp stock solutions to 245 mL distilled water to prepare a 250 mL solution, respectively. The solution mixtures were sprayed directly on the leaves to the point of “drip-off” (forming droplets). The control plants were sprayed with water only. The controls were separated during bio-stimulant spraying to avoid contamination. To avoid nutrient deficiencies, the plants were fertilised/watered with full strength of 1 g/L Hygroponics and 0.8 g/L Solu-Cal (Calcium Nitrate) Ca (NO_3_)_2_ water-soluble nutrient solution every two weeks, using a protocol from Hygrotech [71]. The Hygroponics and Solu-Cal nutrient mixture contained macro- (nitrogen, phosphorus, potassium, calcium, magnesium, sulphur) and micronutrients (Boron, copper, iron, manganese, molybdenum, zinc).

### 4.2. Chlorophyll a Fluorescence and Normalised Difference Vegetation Index (NDVI)

Lightweight leaf clips were used to dark-adapt the leaves for 45 min (one representative leaf per plant). Chlorophyll *a* fluorescence was measured using a Pocket PEA chlorophyll fluorimeter (Hansatech Instrument Ltd., King’s Lynn, UK) by opening and attaching the fluorimeter onto the clip. The fluorimeter automatically calculates the photochemical efficiency of photosystem (PS) II and PSꓲ. In this study, three specific Chlorophyll *a* fluorescence parameters were selected to provide information on the activity of PSꓲꓲ and PSꓲ under specific drought stress conditions: *Fv*/*Fm* ratio (maximum PSII quantum yield), calculated from the following parameters/variables: *Fo* (initial fluorescence), *Fm* (maximum fluorescence), *Fv* (variable fluorescence = *Fm*-*Fo*); performance index absorbance: PI_ABS_ (overall functionality of the electron flow through photosystem ꓲꓲ efficiency); and total performance index: PI_Tota_l (overall functionality of the electron flow from photosystem ꓲꓲ to photosystem ꓲ, thus, total photosynthetic performance). The NDVI values were monitored using a Plant Pen NDVI 310 m (Photon Systems Instruments Ltd., Brno, Czech Republic) on the sample leaf. Measurements were taken between 9:00 a.m. and 12:00 p.m. (weekly) when there was increased light intensity.

### 4.3. Stomatal Conductance

Stomatal conductance was performed using a SC-1 Leaf Porometer (Meter Group, Inc., Washington, DC, USA). Measurements were taken between 10:00 a.m. and 12:00 p.m. (weekly) on the fully expanded mature leaves receiving sunlight on the upper (abaxial) surface of the leaf. A leaf porometer calculates stomatal conductance from the relative humidity gradient [72].

### 4.4. Chlorophyll and Carotenoid Content

The chlorophyll contents (Chlorophyll *a* (Chl *a*), Chlorophyll *b* (Chl *b*) and carotenoids) from frozen spinach leaves were determined according to the method of Lichtenthaler and Miehé [73]. Leaf tissue (100 mg) was crushed in liquid nitrogen and extracted with 5 mL 80% (*v*/*v*) aqueous acetone. The homogenate was centrifuged at 3000× *g* for 5 min at 4 °C, and the supernatant was used to read the absorbance at 663 nm (Chl *a*), 645 nm (Chl *b*) and 470 nm (carotenoids) on Cary 100 Bio (Varian, Sydney, Australia).

### 4.5. Determination of Total Soluble Sugars

Total soluble sugar (TSS) content was measured using a modified method described by Irigoyen et al. [74]. Spinach leaves were oven dried for 72 h at 76 °C to obtain dry tissue. Dried leaves (0.2 g) were extracted in 5 mL ethanol (96%, *v*/*v*). The extract was incubated (80 °C, 10 min) and centrifuged (4000× *g*, 10 min, 4 °C). The supernatant (100 μL) was reacted with (2.9 mL^−1^) anthrone reagent (150 mg anthrone dissolved in 100 mL of 72% (*v*/*v*) sulphuric acid). The reaction mixtures were vortexed and incubated at 80 °C for 15 min. A blue green colour developed, and the tubes were cooled down. The reaction mixtures (3 mL) were vortexed again, and the change in absorbance was measured at 625 nm (Cary 100 Bio, Varian, Sydney, Australia) using plastic cuvettes.

### 4.6. Proline Determination

Proline content was determined using a method described by Carillo and Gibon [75]. A leaf sample (0.3 g) was crushed in liquid nitrogen on ice and mixed with (4 mL) 70% (*v*/*v*) ethanol. The homogenate was centrifuged at 3000× *g* for 10 min at 4 °C, and the supernatant was collected and transferred into a clean test tube. The supernatant (500 μL) was transferred into a 2 mL Eppendorf tube and mixed with (500 μL) 20% (*v*/*v*) ethanol and (500 μL) 1% (*w*/*v*) ninhydrin reagent prepared in 100 mL 60% (*v*/*v*) glacial acetic acid. The mixture was vortexed and incubated (95 °C, 20 min) and allowed to cool down. After cooling, the reaction mixture was centrifuged (10,000× *g*, 10 min). The absorbance was measured at 520 nm (Cary 100 Bio, Varian, Australia) against a blank of 70% (*v*/*v*) ethanol in plastic cuvettes. The free proline was determined using an L-Proline standard curve.

### 4.7. Determination of Electrolyte Leakage

Electrolyte leakage (EL), a measure of membrane stability, was measured according to the method described by Rolny et al. [76]. Ten (10) fresh leaf discs (0.8 cm) were allowed to float in 15 mL deionised water in a test tube, and conductivity was measured afterwards using a conductivity meter (Hanna Instruments (Pty) Ltd., Midrand, SA). This represented an initial (C_0_) electrolyte leakage reading. The leaf discs were then incubated for 3 h at room temperature, and the conductance was re-recorded (C_max_) afterwards. The discs were then boiled in a water bath for 10 min and allowed to cool to room temperature, and the final conductance was recorded (C_tota_l). Electrolyte leakage was calculated as a percentage: % EL = 100 × (C_max_ − C_0_)/C_total_.

### 4.8. Enzyme Extract Preparation, Antioxidative Enzyme and Protein Assays

Enzyme extractions were performed using a modified method of Pukacka and Ratajczak [77]. Frozen leaf material (0.5 g) for each treatment was ground to a fine powder in liquid nitrogen using a pre-cooled mortar and pestle. The ground powder was mixed with 5 mL of the extraction buffer (50 mM potassium phosphate buffer, pH 7.0, containing 0.1% (*v*/*v*) Triton X-100, 2% (*w*/*v*) polyvinylpyrrolidone (PVP), 1 mM ascorbate and 1 mM EDTA). The homogenate was centrifuged (15,000× *g*, 20 min, 4 °C), and the supernatant obtained served as the enzyme extract. All steps were carried out on ice.

Ascorbate peroxidase activity was determined using a modified method of Mishra et al. [78]. The enzyme assay mixture (1 mL) contained 550 μL 50 mM phosphate buffer (pH 7.0), 200 μL 100 mM H_2_O_2_, 150 μL 0.5 mM sodium ascorbate, 50 μL 0.1 mM EDTA and 50 μL enzyme extract. The decrease in absorbance was measured at 290 nm (Cary 100 Bio, Varian, Australia) for 5 min at 20 °C using quartz cuvettes. The ascorbate activity was calculated using an extinction coefficient of 2.8 mM^−1^ cm^−1^.

Guaiacol peroxidase activity was determined using the method of Zieslin and Ben-Zaken [79]. The assay solution (1 mL) contained 500 μL 80 mM phosphate buffer (pH 5.5), 50 μL 200 mM H_2_O_2_, 100 μL 50 mM guaiacol, 340 μL distilled H_2_O and 10 μL enzyme extract. Using plastic cuvettes, the increase in absorbance was measured at 470 nm (Cary 100 Bio, Varian, Australia) for 3 min at 30 °C. The guaiacol peroxidase activity was calculated using an extinction coefficient of 26.6 mM^−1^ cm^−1^.

Glutathione reductase (GR) activity was determined by monitoring the oxidised glutathione (GSSG)-dependent oxidation of NADPH at 25 °C for 3 min at 340 nm (Cary 100 Bio, Varian, Australia), as described by Foyer and Halliwell [80]. The reaction mixture (1 mL) contained 470 μL 100 mM potassium phosphate buffer (pH 7.8), 30 μL 2.0 mM EDTA, 230 μL 0.5 mM oxidised glutathione (GSSG), 230 μL 0.2 mM NADPH and 40 μL enzyme extract. The glutathione reductase activity was calculated using an extinction coefficient of 6.22 mM^−1^ cm^−1^.

The protein content was determined according to the method of Bradford [81] using gamma-globulin as a standard (1.5 mg mL^−1^).

### 4.9. Growth Parameters

Plant/shoot height and root length were measured, and their dry weights were determined, after oven drying at 75 °C (Labotec (Pty) Ltd., Midrand, SA) for five days to a constant weight. In addition, leaf number, leaf surface area (LSA), relative water content (RWC) and stem and root moisture were determined. The leaf numbers were recorded by visually counting the green leaves per pot plant. The LSA and RWC were calculated using 10-leaf discs punched out using an 8 mm diameter cork-borer. The SLA was calculated as

Leaf area = Fresh of all leaves × (surface area × n discs)/Fresh mass of n discs. The RWC was calculated according to González and González-Vilar [82] as RWC (%) = [(FW − DW)/(TW − DW) × 100], where FW represented the initial fresh weight; TW was the turgid fresh weight of discs after being hydrated in distilled water for 24 h in a dark cold room at (4 °C); DW was the dry weight; and RWC was the relative water content. The stem and root moisture contents were calculated as described by Ryser et al. [83] using the equation: Moisture content (%) = 100 − (Dry mass/Fresh mass) × 100.

### 4.10. Statistical Analysis

Data were analysed using a two-way analysis of variance (ANOVA) on GenStat 19th edition (VSN International Ltd., Hertfordshire, UK) [84]. The mean differences between water treatments and bio-stimulant treatments were tested using Tukey’s test at the significance level *p* ≤ 0.05. Pearson’s correlation analysis was performed to determine a relationship between the vegetative, physiological and biochemical parameters under drought stress at different bio-stimulant concentrations using Statistical Analysis System (SAS software for windows version 9.4, Institute Inc., New York, NY, USA) [85].

## 5. Conclusions

Foliar application of *Xcell Boost* effectively increased the performance of different physiological and biochemical parameters, with pronounced effects under drought stress than under optimal irrigation. The most significant impact of *Xcell Boost* on the studied parameters under different water levels was due to the BX2 concentration. Although the effect of *Xcell Boost* differed with the concentration, its application upregulated the photosynthetic capacity of spinach through increased chlorophyll *a* and NDVI under drought stress. It also increased the antioxidative capacity of drought-stressed spinach through increased carotenoids, APX and GPX. Furthermore, the application of *Xcell Boost* on drought-stressed spinach increased the growth responses (leaf surface area, stem dry weight, root dry weight, leaf dry weight, root moisture, leaf number, root length and plant height). Under severe drought stress, the BX2 treatment induced strong positive correlations between stem dry weight, stomatal conductance and APX activity. Moreover, root length positively correlated with total chlorophyll content under BX2. These results suggest that *Xcell Boost* could be used/employed to enhance spinach’s photosynthetic efficiency and biochemical parameters, especially under water-deficit conditions, and positively influence the growth.

## Figures and Tables

**Table 1:** Maximum PSII quantum yield (*Fv/Fm*), performance index absorbance (PI_ABS_) and total performance index (PI_Tota_l) of spinach grown under different water and *Xcell Boost* levels.

WHC (%)	[*Xcell Boost*]	*Fv/Fm*	PI_ABS_	PI_Total_
100	BX0	0.831 ± 0.0099 a	7.48 ± 1.13 ab	6.36 ± 0.58 a
	BX1	0.826 ± 0.0032 a	6.70 ± 0.65 b	6.03 ± 0.35 a
	BX2	0.834 ± 0.0096 a	8.09 ± 0.98 a	6.32 ± 0.75 a
50	BX0	0.836 ± 0.0058 a	9.71 ± 0.5973 a	8.09 ± 0.80 a
	BX1	0.832 ± 0.0067 a	8.77 ± 1.1710 a	7.09 ± 0.66 b
	BX2	0.831 ± 0.0035 a	8.90 ± 0.85 a	7.40 ± 0.62 b
30	BX0	0.838 ± 0.0059 a	8.74 ± 0.53 a	6.95 ± 0.21 a
	BX1	0.831 ± 0.0045 a	7.74 ± 0.90 a	6.20 ± 0.63 a
	BX2	0.832 ± 0.0073 a	7.78 ± 0.54 a	6.28 ± 0.39 a

The values are means of four replicates ± standard error. Letters in the column indicate significant differences within each water treatment at *p* ≤ 0.05. WHC: Water holding capacity. BX0 represents no bio-stimulant (*Xcell Boost*), while BX1 represents single concentration and BX2 double concentration of the bio-stimulant.

**Table 2:** Vegetative growth parameters of *Xcell Boost* treated spinach under water-deficiency stress.

WHC (%)	[*Xcell Boost*]	PH	RL	LA	LN	LDW	SDW	RDW	RWC	SM	RM
100	BX0	23.5 ± 2.1 a	26.5 ± 3.7 a	0.8165 ± 87.18 a	7.5 ± 0.65 a	4.59 ± 0.6 b	5.21 ± 0.69 a	5.3 ± 1.21 a	76.59± 0.82 b	90.02 ± 1.04 a	69.4 ± 1.28 ab
	BX1	24.65 ± 2.2 a	31.7 ± 2.25 a	1365 ± 62.31 a	6.33 ± 0.47 a	5.48 ± 0.58 ab	5.43 ± 0.50 a	5.35 ± 1.31 a	78.31 ± 1.07 b	90.96 ± 0.41 a	71.5 ± 2.79 a
	BX2	23.7 ± 0.53 a	29 ± 4.65 a	1465 ± 79.1 a	8.25 ± 0.75 a	5.86 ± 0.45 a	5.97 ± 0.48 a	6.92 ± 1.22 a	80.29 ± 1.97 a	90.25 ± 1.40 a	65.8 ± 1.78 b
50	BX0	23.4 ± 0.54 a	28. ± 2.16 a	899 ± 65.35 b	7.33 ± 0.8 a	4.39 ± 0.41 a	3.84 ± 0.42 b	7 ± 1.1 a	72.73± 0.89 c	88.94 ± 1.90 a	60.6 ± 2.26 a
	BX1	24.8 ± 2.03 a	32 ± 2.62 a	1290 ± 56.31 a	6.75 ± 0.5 a	4.62 ± 0.47 a	4.54 ± 0.53 ab	7.5 ± 1.2 a	75.4 2± 1.54 b	89.38 ± 1.32 a	56.2 ± 4.1 b
	BX2	22.65 ± 1.65 a	32.25 ± 4.24 a	1321 ± 54.2 a	7.75 ± 0.85 a	5.38 ± 0.4 a	5.23 ± 0.63 a	7.43 ± 1.15 a	78.93± 1.04 a	88.83 ± 0.52 a	62.5 ± 1.02 a
30	BX0	18.4 ± 0.43 b	24 ± 3.6 b	723 ± 17.76 b	7 ± 0.58 a	3.5 ± 0.34 a	3.15 ± 0.76 a	4.92 ± 1.23 a	68.35± 1.98 c	89.75 ± 1.22 a	56.2 ± 1.8 b
	BX1	21.57 ± 1.2 a	27 ± 1.41 ab	799 ± 127.5 ab	6.5 ± 0.625 a	3.78 ± 0.15 a	3.24 ± 0.66 a	5.65 ± 1.2 a	72.6± 0.63 b	89.69 ± 1.26 a	45.6 ± 4.24 c
	BX2	19.3 ± 0.65 ab	32 ± 0.8165 a	843 ± 41.2 a	7.87 ± 0.85 a	4.32 ± 0.39 a	3.41 ± 0.73 a	6.17 ± 1.23 a	76.49 ± 0.69 a	88.11 ± 0.78	61.82 ± 1.93 a

The values are means of four replicates ± standard error. Letters in the column indicate significant differences within each water treatment using Tukey’s test (*p* ≤ 0.05). WHC: water holding capacity, PH: plant height, RL: root length, LA: leaf area, LN: leaf number, LDW: leaf dry weight, SDW: stem dry weight, RDW: root dry weight, RWC: relative water content, SM: stem moisture, RM: root moisture. BX0 represents no bio-stimulant *(Xcell Boost*), while BX1 represents single concentration and BX2 double concentration of the bio-stimulant.

**Table 3:** The Pearson correlation analysis for the photosynthetic capacity, biochemical responses and vegetative growth parameters of *Xcell Boost* treated spinach under severe water deficiency (30% soil WHC) stress.

	LDW	SDW	APX	GR	PI_abs_	PI_total_	SC	NDVI	Chl-*b*	Chl-total	RWC
**RL**	0.99	−0.50	−0.40	0.86	−0.19	−0.77	−0.41	0.80	0.16	0.96	0.35
**LDW**	1.00	−0.63	−0.53	0.90	−0.24	−0.78	−0.54	0.86	0.20	**0.91**	0.49
**SDW**	0.42	1.00	0.99 *	−0.65	0.27	0.56	0.99 *	−0.70	−0.16	−0.28	−0.98 *
**GPX**	−0.98 *	−0.31	0.78	0.43	−0.81	0.35	−0.62	0.53	0.76	−0.35	0.56
**GR**	−0.43	−0.19	0.90	1.00	−0.64	−0.45	−0.58	0.99 *	0.59	0.70	0.50
**PI_abs_**	−0.02	−0.54	0.38	0.75	1.00	−0.40	0.27	−0.69	−0.99 *	0.03	−0.17
**SC**	−0.48	−0.07	0.96 *	0.99 *	0.63	0.78	1.00	−0.65	−0.17	−0.18	−0.99 *
**Car**	−0.19	0.19	−0.43	−0.77	−0.92	−0.97 *	−0.68	0.72	0.03	−0.14	0.96
**Chl-*a***	0.04	−0.02	−0.74	−0.92	−0.78	−0.98 *	−0.89	0.47	0.65	−0.90	0.05
**Proline**	0.10	−0.42	0.36	0.74	0.99 *	0.88	0.62	−0.87	−0.61	−0.47	0.18
**Leaf no**	0.97 *	0.59	−0.64	−0.55	−0.26	−0.01	−0.56	0.26	−0.63	0.84	0.72
**MC leaf**	−0.49	−0.96 *	−0.16	−0.01	0.29	−0.29	−0.11	−0.59	0.59	−0.23	−0.62
**MC root**	0.00	−0.08	0.67	0.90	0.86	0.99 *	0.85	−0.58	−0.66	−0.63	−0.64

Bold represents significance at *p* < 0.05, while bold plus asterisk represents significance at *p* < 0.01. RL = Root length, LDW = Leaf dry weight, SDW = Stem dry weight, RDW = Root dry weight, APX = Ascorbate peroxidase, GPX = Guaiacol peroxidase, GR = Glutathione reductase, SC = Stomatal conductance, Chl-*a* = Chlorophyll a, Chl-*b* = Chlorophyll *b*, Chl-total = Chlorophyll total, Car = Carotenoids, TSS = Total soluble sugars, EL = Electrolyte leakage, RWC = Relative water content, MC stem = Stem moisture content, MC root = Root moisture content. The top-right shaded triangle represents the correlations under double concentration (BX2) *Xcell Boost* treatment. The non-shaded bottom triangle represents the correlations under single concentration (BX1) *Xcell Boost* treatment.

## Data Availability

Not applicable.
